# Psychiatric symptoms in Salla disease

**DOI:** 10.1007/s00787-022-02031-5

**Published:** 2022-07-07

**Authors:** Ida Aulanko, Elisa Rahikkala, Jukka Moilanen

**Affiliations:** 1 PEDEGO Research Unit, University of Oulu, Oulu, Finland; 2 Medical Research Center Oulu, University of Oulu and Oulu University Hospital, Oulu, Finland; 3 Doctoral Programme in Clinical Research, University of Helsinki, Helsinki, Finland; 4 Institute of Biomedicine, University of Turku, Turku, Finland; 5 Department of Clinical Genetics, Oulu University Hospital, OYS, P.O. Box 23, 90029 Oulu, Finland

**Keywords:** Salla disease, Organic psychosis, Sialuria, Sleeping disorder, Behaviour disorder, Register-based study, Clinical report

## Abstract

Salla disease (SD) is a rare lysosomal storage disorder characterised by intellectual disability ataxia, athetosis, nystagmus, and central nervous system demyelination. Although the neurological spectrum of SD’s clinical phenotype is well defined, psychotic symptoms in SD remain unreported. We reviewed the presence of psychiatric symptoms in patients diagnosed with SD. Medical records of all SD patients at Oulu University Hospital during the years 1982–2015 were systematically reviewed to evaluate the presence of psychiatric symptoms. Psychiatric symptoms were frequently associated with SD (10/24, 42%), and two patients were described as developing psychosis as adolescents. We reported their clinical characteristics in detail and assessed the prevalence of psychiatric symptoms in a cohort of 24 patients. Other psychiatric factors associated with SD were sleeping disorders (8/24, 32%), aggressive behaviour disorders or restlessness (6/24, 25%), and off-label antipsychotic medication (4/24, 17%). This report expands the knowledge of the phenotypic spectrum of SD and demonstrates the importance of recognising the possibility of psychiatric symptoms, including psychosis, in persons with SD.

## Background

Salla disease (SD; OMIM 604,369) is a rare autosomal recessive lysosomal storage disorder characterised by sialic acid accumulation in the urine and lysosomes of various tissues [1, 2]. Leading to severe or progressive psychomotor retardation, SD is caused by a homozygous or a compound heterozygous mutation in the *SLC17A5* gene on chromosome 6q13 [3]. It is a mild form of free sialic acid storage disorder (FSASD), and the type of mutation in the *SLC17A5* gene correlates with the severity of the clinical phenotype [4–6]. The *SLC17A5* gene is responsible for lysosomal membrane sialic acid transport and is crucial for normal central nervous system myelination [3, 7, 8]. Magnetic resonance imaging (MRI) studies of people with SD have revealed several typical central nervous system changes, including cerebral and cerebellar atrophy, dysmyelination, and corpus callosum hypoplasia [9–11].

SD belongs to the so-called Finnish disease heritage, although cases with molecularly proven SD have been detected elsewhere in the world [4, 12–14]. Two phenotypes of SD, conventional, and severe, have been described based on the clinical phenotype [15]. More than 90% of Finnish SD patients are homozygous for the pathogenic *SLC17A5* c.115C > T; p.(Arg39Cys) (NM_012434.5) founder variant associated with the milder phenotype of the disease.

The clinical phenotype of SD varies widely, ranging from mildly affected individuals who can speak and walk unassisted to non-ambulatory patients with severe intellectual disability (ID) [10, 15]. SD is usually diagnosed in early childhood. The first symptoms are typically detected between the ages of 3–12 months. Ataxia, delayed motor development, nystagmus, and muscular hypotonia are the most common early symptoms [2, 15]. Athetotic movements, spasticity, and epilepsy become more prominent as people with SD age. All SD patients have ID, and the disease appears to slightly decrease life expectancy [2, 12].

Previous studies have highlighted the overrepresentation of people with ID in psychiatric patients and the common off-label antipsychotic medication in persons with ID [16, 17]. To our knowledge, the prevalence of psychiatric symptoms in SD remains unreported. The majority of SD patients live in northern Finland; therefore, Oulu University Hospital provides an innate location for studying SD.

We report a register-based analysis of psychiatric symptoms associated with SD and, in detail, two patients, a male and a female, with SD and early-onset psychosis [18].

## Methods

The study was conducted as a retrospective register-based study. We identified 24 people diagnosed with Salla disease (SD) from the registry of Oulu University Hospital Department of Clinical Genetics (from 1982 to 2015). The patient charts of all identified individuals were systematically reviewed for psychiatric symptoms and medications. Patient identification was based on diagnostic codes according to the International Classification of Diseases (ICD-9 and ICD-10). Psychiatric symptoms were first identified by identifying patients with patient records from a psychiatric clinic or described psychiatric medication. The patient charts of these individuals were examined more thoroughly by two independent authors to identify symptoms and clinical phenotypes. Statistical analysis was conducted using R Statistical Software (version 1.3–0; R Foundation for Statistical Computing, Vienna, Austria).

The median age of the patients was 21 years, ranging from 6 to 63. Children or young adults under 20 years old represented 41.7% (10/24) of the cohort. Those aged between 20 and 40 comprised 37.5% (9/24). Only five individuals (5/24, 20.8%) were aged 40 and older. A majority of the patients, 75% (18/24), were male, and 25% (6/24) were female.

## Results

We identified two SD patients (Patients 1 and 2) who were described in psychiatric records as developing psychotic symptoms during adolescence, which relapsed whenever antipsychotic medications were discontinued. The clinical phenotypes of the patients are described by the information that was available. Hallucination and insomnia were the main psychiatric symptoms of both reported patients. Both patients responded to antipsychotic medication and required long-term medication. Discontinuation of antipsychotic medication resulted in a relapse of psychosis.

One in four (6/24) individuals were on antipsychotic medication for behavioural problems associated with ID. Off-label usage of antipsychotic medication occurred in 4/24 (17%). Besides the two patients discussed in detail, one patient, on the verge of puberty, had a five-month lasting episode of restlessness, confusion, insomnia, and abnormal behaviour. The patient seemed to be looking for someone continuously. The patient’s symptoms resolved with levomepromazine, and the medication was discontinued, with no relapse of the symptoms. Medications prescribed included perazine, pericyazine, thioridazine, risperidone, levomepromazine, and chlorprothixene. Other psychiatric symptoms associated with SD were sleeping disorders (8/24, 33%) and aggressive behaviour disorders or restlessness (6/24, 25%). Aggressive behaviour included, for example, episodes of screaming, hitting people, and pulling things.

### Patient 1

Patient 1 was a 43-year-old Caucasian woman diagnosed with SD at the age of five. She was born at term after an uneventful pregnancy to non-consanguineous parents. Her birth and early development were normal. Ataxia was noted at the age of one year. Her speech and psychomotor development were delayed. Strabismus and tremors were noted at the ages of four to five. At the age of five, urine oligosaccharide analysis showed repeatedly raised urine sialic acid levels, confirming the clinical suspicion of SD.

The patient has severe IDs, and at the age of 23, her developmental age corresponds to the level of a 4.5-year-old. She needs help in all essential daily activities (eating, getting dressed, and personal hygiene). The patient has learned to speak in short sentences of two to three words, and she walks unassisted. She has no history of seizures but suffers fluctuating ataxia, strabismus, progressive clumsiness, and muscular hypotonia. Her fine motor skills are clumsy, and she has distal rigidity. She has thoracal kyphosis and strabismus. Her psychiatric condition has been prescribed as fluctuating throughout her life.

The patient’s initial psychotic symptoms occurred at the age of 11. During the initial symptoms and later relapses, she has been described as disoriented, having difficulties recognising familiar people, sleeping problems, and mood swings. Visual hallucinations and restless but not aggressive behaviour have been described in the patient’s records without any further details. During the initial psychotic symptoms, the patient’s abnormal behaviour was noticed by both her parents and their neighbour. An electroencephalogram (EEG), showing a mild diffuse slowing of background activity, was taken before medicating her with diazepam and chloral hydrate, which quickly relieved her symptoms within hours.

The patient’s sleeping problems and psychotic symptoms have been treated with several medications and combinations of medications over the years. These have included diazepam, temazepam, perazine, zopiclone, buspirone, hydroxyzine, risperidone, quetiapine, zolpidem, melatonin, and alprazolam. The patient’s symptoms have been considered psychotic in the patient records by several doctors.

Several attempts to discontinue the patient’s antipsychotic medication due to side effects or to the patient’s or parents’ wishes have occurred. While not on antipsychotic medication, benzodiazepines have been used for restlessness and sleeping problems. Within one to three years of discontinuation of the antipsychotic medication, the patient suffered from a psychotic relapse. The patient had a favourable response to perazine and quetiapine. While on risperidone, the patient had been in a stable psychiatric condition for almost 10 years until she became restless, and her medication was changed to quetiapine.

No additional descriptive details on the diagnosis of psychosis are available. Therefore, the evidence for psychosis must be considered tentative, although suggested by years of psychiatric treatment records that conclude that psychosis was present initially and relapsed when antipsychotic medication was stopped.

### Patient 2

Patient 2 was a 22-year-old Caucasian man diagnosed with SD at the age of 1.5. He was born at term after an uneventful pregnancy to non-consanguineous parents. His birth and early development were normal. At the age of one, hypotonia and tremors were noticed. A brain MRI revealed an almost complete absence of myelination typical of SD. DNA analysis revealed a homozygous *SLC17A5* c.115C > T; p.(Arg39Cys) founder variant in the *SLC17A5* gene, confirming the clinical suspicion of SD. Both of his parents were heterozygous carriers of this variant.

In the psychological assessment at the age of 16, his cognitive skills were estimated to correspond to a three- to four-year-old with severe ID. The patient has a cheerful personality. He understands simple speech, communicates mainly using sign language, and has a vocabulary of a few words. He can walk unassisted. The individual has ataxia, clumsiness, and no history of seizures.

Psychotic symptoms started at the age of 14. The first symptoms noted by the patient’s parents were auditory hallucinations combined with absentmindedness, impatience, and anxiousness. The patient had difficulties with orientation, flinches, trouble recognising familiar people, and problems in eating and sleeping. The patient had fears of someone breaking in and told his mother that he had heard voices. In a psychiatric evaluation, disunity of mind, dependency, and trouble getting in contact with the patients were noticed after a week of psychosis. According to the patient’s records, the psychiatrist and paediatric neurologist considered the patient’s symptoms psychotic. Symptoms were significantly reduced within days after the commencement of antipsychotic medication in a psychiatric ward. The patient experienced several emotional stress factors when the symptoms started. He was at the onset of puberty and had several changes in his daily life as he changed school.

The patient was initially treated with levomepromazine, which was soon replaced by risperidone. Antipsychotic medication was reduced stepwise and terminated after gaining a stable psychiatric condition, but was started again within two months from discontinuation due to a psychotic relapse. The patient was again restless and had trouble eating, sleeping, and concentrating on things he usually enjoys. He appeared to be hearing voices others did not hear and was picking up his ears as if trying to listen more carefully to something. He acted abnormally, for example, by continuously walking around, knocking on walls, breaking things at home, and emptying closets. The patient responded well to treatment with risperidone and had a stable psychiatric condition for several years. In addition to antipsychotic medication, oxazepam was occasionally used for sleeping problems and anxiety. Regarding the side effects of the medication, the patient suffered from tiredness and excessive salivation. No additional descriptive details of the diagnosis of psychosis are available; therefore, the evidence for psychosis must be considered tentative. However, years of psychiatric treatment records suggest that psychosis was present initially and relapsed when antipsychotic medication was stopped.

At the onset of psychosis, the patient was screened thoroughly for somatic causes of the symptoms by undergoing a brain MRI, EEG, and electrocardiogram (ECG). The EEG showed mild diffuse background slowing, and the MRI revealed an almost complete absence of myelination. Routine laboratory tests were obtained: low blood count and blood levels of alanine aminotransferase, cortisol, glucose, potassium, sodium, creatinine, C-reactive protein, thyroid-stimulating hormone, and free thyroxine were normal. The venous blood gas analysis was normal, the screening for toxins and drugs in the urine was negative, and the urine culture was sterile.

## Discussion

This study reports on two individuals with Salla disease (SD) who developed psychosis during their teenage years, suggesting that psychosis might be associated with SD. We identified 24 individuals in Northern Finland with an SD diagnosis and recent data on their health. Other psychiatric symptoms associated with SD in our study were sleeping disorders and aggressive behaviour or restlessness.

Based on information from psychiatric records, the prevalence of psychosis among people with SD was 8.3% (2/24), which is higher than the prevalence of psychotic disorders among individuals with ID in general (4.4%) [16, 19], but the difference is not significant (*p* = 0.29, two-sided exact binomial test). Our case series represents an unusually large collection of SD patients, but a proportion power calculation for binomial distribution indicates that with 24 cases, the statistical power to detect an effect of the observed magnitude at the significance level of 0.05 is still only 13%, and a power of 80% would require a many times larger number of cases, which is currently unattainable due to the rarity of SD. Therefore, our study confirms that psychotic symptoms are part of the clinical spectrum of SD and suggests that their frequency is similar to other severe ID and may be higher. Based on this initial series, it remains difficult to determine whether psychotic symptoms are specifically elevated in SD, or whether they should be tentatively viewed as a non-specific effect of severe ID.

Psychotic disorders, including schizophrenia, usually begin in adolescents or young adults [20–22]. Due to the low median age of 21 (ranging from 6–63) in our sample, some persons may develop psychiatric symptoms or even psychosis in the future. Of this cohort of 24 individuals, 15 were under 30 years old.

In addition to the two SD patients diagnosed with psychosis, four out of the 24 patients in this cohort had been treated with antipsychotic medication due to sleeping problems, restlessness, aggressiveness, and behavioural problems. Psychotropic medication is common among individuals with ID, even when they are not diagnosed with a psychiatric disease [17]. Previous studies have revealed that up to 49–74% of people with ID have a prescription for antipsychotic medication, and recent studies have questioned the practice of widespread off-label antipsychotic medication [17, 23].

To the author’s knowledge, no previous cases of early onset psychosis in individuals with SD have been reported. The psychiatric problems of one person have been mentioned in studies concerning the phenotypic spectrum of SD (15/24). Both patients described in this article were on the verge of puberty, and one of them had emotional stress factors at the onset of psychotic symptoms. Puberty, other stressful life events, and SD were possibly contributing factors in the onset of psychotic symptoms. The symptoms could not be explained by familial susceptibility to psychosis, because neither of the patients had a family history of psychiatric disorders. It is important to note that both individuals required long-standing antipsychotic medication, and discontinuation of the medication resulted in a relapse of psychosis. Both individuals responded to antipsychotic medication.

Diagnostics of psychosis can be challenging, especially in severely intellectually disabled people. Diagnostic tools (ICD-10-DCR, DSM-IV-TR), commonly applied to the general population, underestimate psychiatric diseases in people with ID [19]. We thoroughly examined all available medical records of individuals with SD between 1982 and 2015 for this study. Both patients described in detail had hallucinations, and their behaviour was disorganised and consistent with the diagnosis of psychosis or psychotic disorder not otherwise specified according to the DSM-IV.

A noticeable proportion of the patients suffered from sleeping problems or behavioural problems (10/24), and some required antipsychotic medication to treat the symptoms (6/24). Medication for sleeping problems and behavioural problems might hide some of the milder cases of psychosis, and psychotic symptoms might very well be more common among this group of people than previously understood.

In conclusion, this study expands the knowledge of the clinical spectrum of SD and suggests that psychiatric symptoms may be more common in people with SD than previously assumed. Healthcare practitioners should consider the possibility of psychosis and the need for antipsychotic medication when treating people with SD.
